# PD93 Budget Impact And Sustainability Of Development Partnerships In The Incorporation Of Antiretrovirals Into The Brazilian Unified Health System

**DOI:** 10.1017/S0266462325102195

**Published:** 2025-12-29

**Authors:** Adson José Moreira, Álex Brunno do Nascimento Martins, Bárbara Rodrigues Alvernaz dos Santos, Ludmila Peres Gargano, Francisco de Assis Acurcio, Augusto Afonso Guerra Júnior, Juliana Alvares-Teodoro

## Abstract

**Introduction:**

The preferred regimen for initiating HIV/AIDS treatment, according to the Clinical Protocol and Therapeutic Guidelines (PCDT), is tenofovir, lamivudine, and dolutegravir (TDF/3TC/DTG). Fiocruz, through a productive development partnership (PDP), produces these antiretrovirals, strengthening the national industry. The National Committee for Health Technology Incorporation (CONITEC) evaluated the budgetary impact of incorporating bictegravir, emtricitabine, and tenofovir alafenamide (BIC/FTC/TAF) combination therapy and its effect on the sustainability of the PDP.

**Methods:**

The analysis used data from the 2022 HIV Clinical Monitoring Report to identify the eligible population. The unit cost of the current regimen (TDF/3TC/DTG) was obtained from the Health Price Database, while the cost of the new regimen (BIC/FTC/TAF) was extracted from CONITEC Recommendation Report No. 675/2021. Using a market share analysis, a moderate 10 percent annual increase in uptake of BIC/FTC/TAF was projected, reaching 50 percent by the fifth year.

**Results:**

For the budget impact calculation, a total of 245,923 people aged 50 years or older who were living with HIV and/or AIDS and undergoing antiretroviral therapy were considered. The costs with and without the incorporation of the BIC/FTC/TAF regimen were analyzed. The incorporation of the new technology resulted in an estimated budget impact of BRL15,463,638.24 (USD2,693,122.18) over the analyzed period.

**Conclusions:**

The BIC/FTC/TAF regimen is an effective, safe treatment approved by international agencies. It is administered in a daily dose, which simplifies treatment. However, its patent restricts national production, and its use in place of the current regimen could impact PDPs, harming the national industry and the sustainability of the health system. Therefore, incorporations require balancing clinical benefits with innovation and financial impacts.

